# District Effect Appraisal in East Sub-Saharan Africa: Combating Childhood Anaemia

**DOI:** 10.1155/2019/1598920

**Published:** 2019-11-13

**Authors:** Danielle J. Roberts, Temesgen Zewotir

**Affiliations:** School of Mathematics, Statistics and Computer Science, University of KwaZulu-Natal, Durban, South Africa

## Abstract

**Background:**

Anaemia in children is a significant health problem that receives little attention. This study aimed at determining the factors significantly associated with anaemia in children aged 6 to 59 months in Kenya, Malawi, Tanzania, and Uganda while accounting for the spatial heterogeneity within and between the districts of the four countries. In addition, the performance of the districts with regard to their impact on anaemia was assessed and ranked.

**Methods:**

A generalised additive mixed model with a spatial effect based on the geographical coordinates of the clusters was used. A district-level random effect was included to further account for the heterogeneity as well as to rank the performance of the districts based on the best linear unbiased prediction (BLUP).

**Results:**

The results depicted significant spatial heterogeneity between and within the districts of the countries. After accounting for such spatial heterogeneity, child-level characteristics (gender, malaria test result, and mother's highest education level), household-level characteristics (household size, household's wealth index Z-score, the type of toilet facility available, and the type of place of residence), and the country of residence were found to be significantly associated with the child's anaemia status. There was a significant interaction between the type of place of residence and the country of residence. Based on the BLUP for the district-level random effect, the top 3 best- and worst-performing districts within each country were identified.

**Conclusion:**

The ranking of the performance of the districts allows for the worst-performing districts to be targeted for further research in order to improve their anaemia control strategies, as well as for the best-performing districts to be identified to further determine why they are performing better and then to use these districts as role models in efforts to overcome childhood anaemia.

## 1. Introduction

Identifying significant factors associated with an increased risk of anaemia in children is relevant to developing appropriate and effective interventions. Such studies aid in identifying subpopulations that are most at risk, which assists in creating a more efficient delivery system of limited national resources [1]. However, studies identifying these factors should account for spatial heterogeneity and spatial autocorrelation in the observations. Failure to do so may produce inaccurate estimates and thus misleading results and ineffective anaemia control programs [2, 3].

Spatial autocorrelation arises when observations close in proximity tend to be more alike than those further apart and is present even if the observations have been recorded in a standardised way [4]. Spatial heterogeneity refers to the spatial variation or uneven distribution of attributes across a region [5]. Climatic and environmental factors, such as temperature, rainfall, and proximity to waterbodies, among others, are largely responsible for such spatial heterogeneity as its effects are usually only partially explained by the covariates that are available in a model [4]. Indeed, many other factors that vary geographically can also contribute to spatial heterogeneity in observations, such as the availability and distance to quality child health care, access to a reasonable transport system, culture, and the cost of living, all of which may not always be fully explained by the available covariates. Various methods of accounting for spatial autocorrelation and spatial heterogeneity have been well established due to the increased accessibility of spatially indexed data [4, 6, 7].

There have been a considerable number of studies assessing the risk factors and determinants of anaemia in children ([8–11] and references therein), some of which have also assessed the spatial variation of anaemia [2, 12–14]. However, few studies have focused on countries in eastern sub-Saharan Africa which experiences a high burden of childhood anaemia [15].

This study aims at determining the factors significantly associated with anaemia in children aged 6 to 59 months in Kenya, Malawi, Tanzania, and Uganda while accounting for the spatial heterogeneity within and between the districts of the four countries. In addition, as local health matters are planned at district level in these countries, the performance of the districts is considered in order to assess and rank the “best” and “worst” performing districts with regard to their impact on childhood anaemia based on the best linear unbiased prediction (BLUP) technique. Such a district effect appraisal on anaemia will aid in providing a wider and richer insight in the effort to overcome childhood anaemia by prioritising the worst-performing districts for action. Furthermore, it enables one to identify key differences between the best- and worst-performing districts compared to national and global levels. Identifying factors that contribute to these differences can aid in targeting the correct set of interventions in the districts where it is much needed. This BLUP technique is primarily used in animal and plant breeding for estimating and ranking genetic merit [16–18]; however, to our knowledge, such a method has not been used for the appraisal of administrative levels in epidemiological studies. Individual studies on childhood anaemia have been carried out in Kenya [19], Malawi [6, 13], Tanzania [20], and Uganda [9]. All these studies differ in scope and coverage. The advantage of focusing on multiple countries that form contiguous regions is to be able to investigate the spatial heterogeneity of childhood anaemia between the countries. This assists in determining whether the significant drivers of childhood anaemia are country specific or whether they cross the borders of the countries and are thus shared between neighbouring countries.

## 2. Materials and Methods

### 2.1. Study Area and Data

This study utilised nationally representative Demographic and Health Surveys (DHS) and Malaria Indicator Surveys (MIS) from Kenya, Malawi, Tanzania, and Uganda, in particular, the 2015 Kenya Malaria Indicator Survey (KMIS2015), the 2017 Malawi Malaria Indicator Survey (MMIS2017), the 2015–2016 Tanzania Demographic and Health Survey and Malaria Indicator Survey (TDHS2015), and the 2016 Uganda Demographic and Health Survey (UDHS2016). The surveys were a national sampling design of stratified two-stage cluster sampling. The geographical coordinates of the sampled clusters, which made up the primary sampling units, were recorded during all the surveys. However, in order to ensure confidentiality of the respondents, the coordinates were randomly displaced, although the region and type of residence (urban/rural) of the coordinates were maintained [21]. Three questionnaires were carried out in the sampled households in order to collect information regarding the household characteristics and eligible men and women. Furthermore, all children under the age of five years in the sampled households were tested for malaria and anaemia with the consent of a parent or guardian. The final dataset consisted of 18247 children from 1595 clusters with valid geographical coordinates within 370 administrative areas, which consisted of all 47 counties or districts for Kenya; 26 out of 28 districts for which data were available for Malawi; 176 out of 184 districts for which data were available for mainland Tanzania; and 121 out of 122 districts for which data were available for Uganda. These administrative levels were chosen based on the levels for which public health decisions are made within each country.

### 2.2. Study Variables

A portable HemoCue analyser was used to measure the haemoglobin (Hb) concentration in the sampled children's finger- or heel-prick blood specimens. Based on the altitude adjusted Hb levels and the World Health Organization (WHO) definitions for anaemia in children aged 6 to 59 months [22], the outcome variable for the *k*^th^ child in the *j*^th^ household within the *i*^th^ cluster and *h*^th^ district was dichotomised as

(1) $$\begin{array}{c} y_{hijk}=\left\{\begin{array}{cc} 1, & \text{if Hb level}<11\;\text{g}/\text{dl}\left(\text{anaemic}\right),\\ 0, & \text{if Hb level}\geq 11\;\text{g}/\text{dl}\left(\text{nonanaemic}\right). \end{array}\right. \end{array}$$

**Figure 1:**
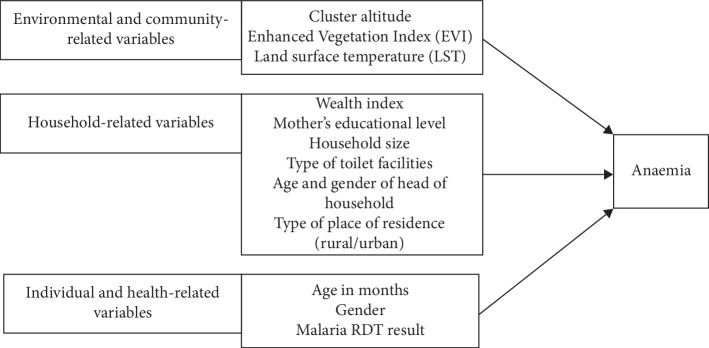
Potential risk factors of childhood anaemia.

The risk factors considered in this study comprise individual-, household-, and community-level variables given in Figure 1. These variables included gender and age of the child, the child's malaria rapid diagnostic test (RDT) result, the mother's highest education level, the number of members in the household (size of the household), the type of place of residence (rural or urban), the cluster altitude, the household's wealth index Z-score, the type of toilet facility, the age and gender of the head of the household, as well as the country of residence (Kenya, Malawi, Tanzania, or Uganda). Furthermore, two environmental factors, average day land surface temperature (LST) and the average Enhanced Vegetation Index (EVI) for 2015, were also considered as they serve as proxies for intestinal parasites, which is a risk factor for childhood anaemia [23–25]. The values of these environmental factors were extracted based on the geographical coordinates of the clusters, and thus, they are regarded as cluster level.

### 2.3. Statistical Methods

We adopted generalised additive mixed models (GAMMs) for the hierarchical and spatially correlated data [26]. GAMM is an additive extension of generalised linear mixed models and uses additive nonparametric functions to model covariates and geospatial effects while accounting for correlation by adding random effects to the additive predictors [27, 28]. The fitted GAMM for *P*(*Y*_*hijk*_=1)=*π*_*hijk*_ with a logit link function is as follows:

(2) $$\begin{array}{c} \log\text{it}\left(\pi_{hijk}\right)=x_{hijk}^{\prime }\beta +U_{h}+\overset{p}{\underset{r=1}{\sum }}f_{r}\left(z_{hijk}\right)+f_{\text{spat}}\left({\text{lon}}_{i},{\text{lat}}_{i}\right), \end{array}$$

where **β** is the linear fixed effects and *U*_*h*_, *h*=1,…, 370, is the district-level random effect modelled parametrically; *f*_*r*_(·),  *r*=1,…, *p*, are the unknown smooth functions that represent the nonlinear effects of the *p* covariates which are modelled nonparametrically, and the nonlinear term *f*_spat_(lon_*i*_, lat_*i*_) is a function of the geographical coordinates of the *i*^th^ cluster where lon_*i*_ and lat_*i*_ are the longitude and latitude, respectively.

Estimation of the smooth functions *f*_*r*_ was based on penalised splines (P-splines) [29]. This approach assumes that the unknown functions can be approximated by a polynomial spline of degree *l* with equally spaced knots *z*_*r*_^{min}^ < *ζ*_*r*0_ < *ζ*_*r*1_ < …<*ζ*_*rn*_*r*_−1_ < *ζ*_*rn*_*r*__=*z*_*r*_^{max}^ which are within the domain of the covariate *z*_*r*_. The spline can be written in terms of a linear combination of *M*_*r*_=*n*_*r*_+*l* B-spline basis functions, *B*_*rm*_, and regression coefficients *α*_*rm*_ as follows:

(3) $$\begin{array}{c} f_{r}\left(z_{r}\right)=\overset{M_{r}}{\underset{m=1}{\sum }}\alpha_{rm}B_{rm}\left(z_{r}\right). \end{array}$$

The choice in the number of knots is important as too few results in a spline that may not be flexible enough to capture the variability in the data; however, too many knots may result in estimated curves that overfit the data, which leads to functions that are too rough [30]. To overcome this problem, a moderately large number of equally spaced knots of between 20 and 40 are used to ensure flexibility [29]. In addition, a roughness penalty is defined based on first- or second-order differences of adjacent B-spline coefficients which guarantee sufficient smoothness of the fitted curves [29]. This leads to penalised likelihood estimation with penalty terms given by

(4) $$\begin{array}{c} P\left(\lambda_{r}\right)=\frac{1}{2}\lambda_{r}\overset{M_{r}}{\underset{m=v+1}{\sum }}{\left(\Delta^{v}\alpha_{rm}\right)}^{2},\;v=1,2, \end{array}$$

where *λ*_*r*_ is the smoothing parameter and Δ^*v*^ is the differencing operator of order *v*. First-order differences penalise abrupt jumps *α*_*rm*_ − *α*_*r*,*m*−1_ between successive parameters, while second-order differences penalise deviations from the linear trend 2*α*_*r*,*m*−1_ − *α*_*r*,*m*−2_ [30]. In this study, a choice of 20 knots was used.

The effect of the *i*^th^ cluster location, given by *f*_spat_(lon_*i*_, lat_*i*_), *i*=1,…, 1595, was estimated based on a two-dimensional P-spline, which itself is based on the tensor product of one-dimensional B-splines:

(5) $$\begin{array}{c} f_{\text{spat}}\left({\text{lon}}_{i},{\text{lat}}_{i}\right)=\overset{1595}{\underset{m_{1}=1}{\sum }}\overset{1595}{\underset{m_{2}=1}{\sum }}\alpha_{m_{1}m_{2}}B_{m_{1}}\left({\text{lon}}_{i}\right)B_{m_{2}}\left({\text{lat}}_{i}\right). \end{array}$$

The stochastic formulation of *f*_spat_(lon_*i*_, lat_*i*_) represents the realisation of a spatially correlated stochastic process, which assists in accounting for spatial correlations in the data [4]. The B-spline basis functions are now spatially aligned along the *x*- and *y*-axes, and thus, a suitable difference penalty is then constructed based on squared deviations of *α*_*m*_1_*m*_2__ from the regression coefficients of the four nearest neighbours [4].

Furthermore, in order to account for the correlation in the responses due to unmeasured district-specific factors, an independently and identically distributed random effect was included in the model based on the district in which the child resided. This is represented by the term *U*_*h*_ in the model given in equation (2). This function for the random effect can also be approximated by a linear combination of B-spline basis functions given in equation (3). However, the regression coefficients *α*_*rm*_ are i.i.d. random effects [30].

GAMMs can be represented as GLMMs after appropriate reparameterisations of the smoothing splines [30]. Based on the GLMM representation, regression parameters and variance components can be estimated using iteratively weighted least squares (IWLS) and restricted maximum-likelihood (REML) estimation, respectively. The mixed-model methodology permits the estimation of the fixed effects, as well as the prediction of the random effects using the BLUP procedure by solving a generalized form of mixed-model equations [31]. BLUP values are realised values of the random effect [32, 33]. BLUP provides an unbiased method by adjusting for known sources of individual, household, cluster, geospatial, and environmental variation [34]. Furthermore, BLUP is an appropriate technique for the ideal ranking or selection criteria that involve a random effect. It is well established on theoretical grounds that these properties can result in increased accuracy in ranking and selection [16, 34, 35]. In other words, the ranking of the best linear predictors produces the same order as the true values of the random effects [36]. Thus, inclusion of the district-level random effect enables one to rank and select the “best” and the “worst” performing districts with regard to the odds of anaemia based on the obtained BLUP estimates for each district.

The estimation approach used in this study is referred to as an empirical Bayes approach. Empirical Bayes inference assumes that the regression and variance parameters are unknown constants, where the estimates are obtained by maximising an objective function. Thus, the usual questions about convergence of MCMC samples or sensitivity on hyperparameters do not arise and the estimates can be interpreted as penalised likelihood estimates from a frequentist perspective [30]. The model was fitted using the R2BayesX package in R [37]. The estimates of the district-level random effect (the BLUP estimates for each district) and cluster-level spatial effect were imported into ArcGIS 10.6 and mapped.

## 3. Results

The final dataset of 18027 children was made up of 3389 (18.8%) children from Kenya, 2271 (12.6%) from Malawi, 7747 (43.0%) from Tanzania, and 4620 (25.5%) from Uganda. The overall observed prevalence of anaemia was 52.5% with lowest in Kenya at 38.30%, and the other countries having a higher observed prevalence ranging from 52.53% to 58.26%.

The age of the head of household was not significantly associated with a child's anaemia status at a 10% level of significance in a univariate analysis and therefore was the only variable not entered into the fitted GAMM. Out of the continuous covariates considered in this study (child's age in months, household size, household wealth index Z-score, cluster altitude, EVI, and LST), only the child's age in months displayed a significant nonlinear effect on the log-odds of anaemia; thus, it was the only nonlinear effect incorporated into the GAMM, with the rest of the covariates entered into the model as linear fixed effects. To avoid possible confounding effects, all two-way interactions of the fixed effects were explored. The only significant interaction was found between the type of place of residence (rural/urban) and the country, which is not a surprising result as the coverage and classification of rural/urban areas within a country differ from country to country. This significant interaction effect suggests that the effect that an urban or rural area has on anaemia in children differs across the four countries. Furthermore, the total effect that the place of residence and country has on the odds of anaemia is made up of their individual main effects as well as the simultaneous/interaction effect between the two variables.

Table 1 displays the results of the adjusted odds ratios and their 95% confidence intervals for the final model. Female children had significantly lower odds of anaemia compared to males (AOR = 0.876; 95% CI: (0.820, 0.935)). The odds of anaemia were significantly higher for children who tested positive for malaria based on the RDT result compared to those who tested negative (AOR = 4.315; 95% CI: (3.895, 4.781)). The odds of anaemia increased with an increase in household size (AOR = 1.014; 95% CI: (1.003, 1.025)); however, the odds decreased with an increase in the household wealth index Z-score (AOR = 0.847; 95% CI: (0.797, 0.901)). Children whose mother had at least a primary level of education were associated with lower odds of anaemia compared to those whose mother had no education (AOR = 0.843; 95% CI: (0.760, 0.935) for primary level; AOR = 0.794; 95% CI: (0.693, 0.911) for secondary or higher education level). Moreover, children in households with improved toilet facilities had lower odds of anaemia compared to those in households with no toilet facilities (AOR = 0.780; 95% CI: (0.694, 0.877) for PIT latrine; AOR = 0.725; 95% CI: (0.591, 0.889) for flush toilet). The gender of the head of household, cluster altitude, EVI, and LST were not significantly associated with the child's anaemia status.

**Figure 2:**
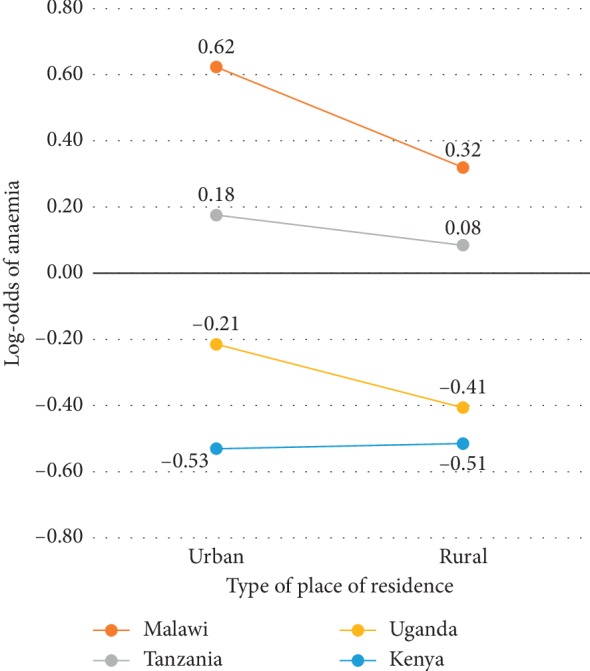
Log-odds of anaemia associated with the type of place of residence and country.

While the adjusted odds ratios for the main and interaction effects of the type of place of residence and country of residence are presented in Table 1, they cannot be interpreted separately. Rather, their total effect on the log-odds of anaemia should be considered. Thus, Figure 2 presents the total estimated log-odds of anaemia for each type of place of residence across the four countries. This figure clearly displays a difference in the effect of the type of place of residence on the log-odds of anaemia between the four countries. Without the inclusion of this interaction effect, it would be assumed that the effect of the type of place of residence is constant for all the countries. While the log-odds of anaemia for children residing in rural areas were lower than that for children residing in urban areas in Malawi, Tanzania, and Uganda, only Malawi displayed a considerable difference between urban and rural areas. Furthermore, Uganda and Kenya displayed decreased log-odds of anaemia in both urban and rural areas, while Malawi and Tanzania displayed increased log-odds in both urban and rural areas.

**Figure 3:**
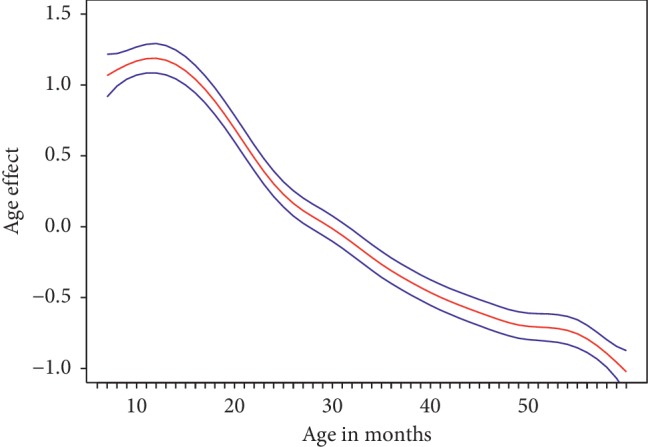
Estimated nonlinear effect of the child's age in months on the log-odds of anaemia together with the 95% confidence interval.

The child's age in months had a fairly significant nonlinear effect on the log-odds of anaemia with its nonzero variance estimate (Table 2). Similarly, the variance estimates for the district-level random effect and cluster-level spatial effect were nonzero.  Figure 3 displays the nonlinear effect of the child's age in months on the log-odds of anaemia. The effect increased from 6 to 10 months of age, after which there was a decline in the effect. Children from about 25 months of age displayed a negative effect and thus were associated with decreased odds of anaemia.

**Figure 4:**
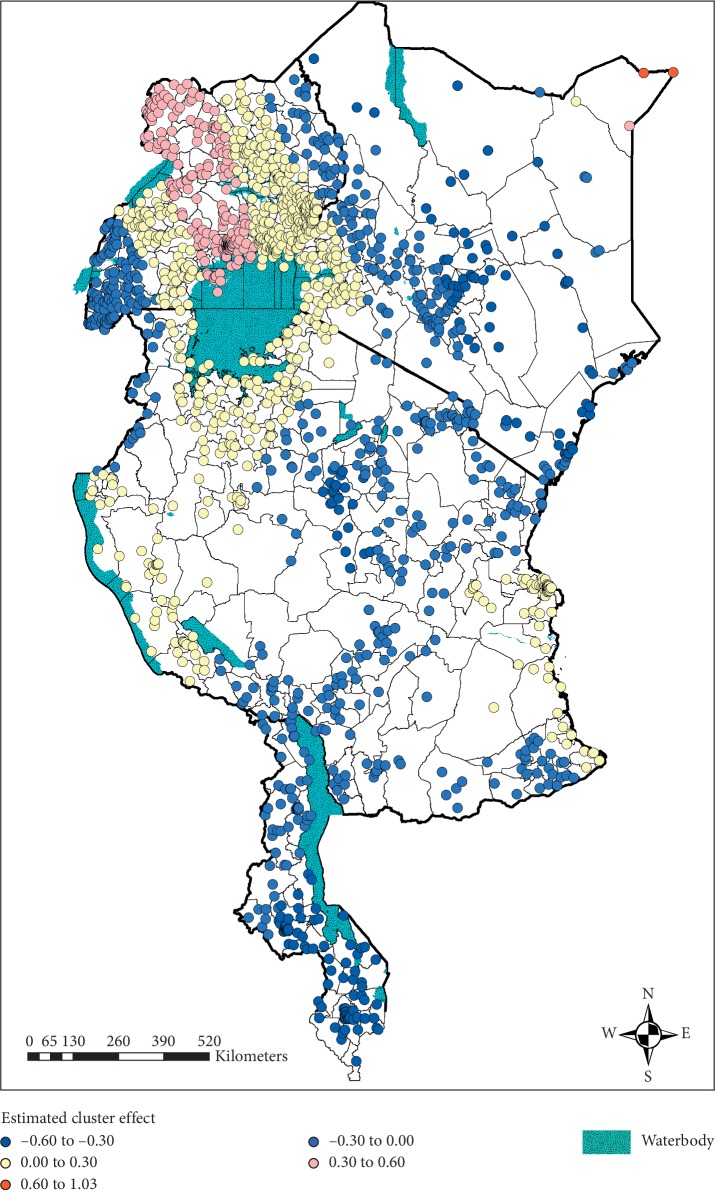
Estimated cluster-level spatial effect on the log-odds of anaemia (top left: Uganda; top right: Kenya; middle: Tanzania; and bottom: Malawi).

The estimated cluster-level spatial effect, which accounts for spatial autocorrelation, is presented in Figure 4. The clusters in shades of blue had a negative effect on the log-odds of anaemia and thus were associated with a decreased risk, whereas those in shades of yellow to red had a positive effect and were therefore associated with an increased risk of childhood anaemia. Uganda, which consisted of clusters with both positive and negative effects, displayed the largest spatial variation. Throughout all four countries, the majority of neighbouring clusters resulted in similar effects. In Kenya, Tanzania, and Uganda, some areas displayed clusters with a positive effect and clusters with a negative effect within the same district. Clusters surrounding Lake Victoria, which lies across the border between Uganda and Tanzania, had a positive effect and thus were associated with increased odds of anaemia. Malawi was fairly homogeneous as it consisted of clusters with only negative effects.

**Figure 5:**
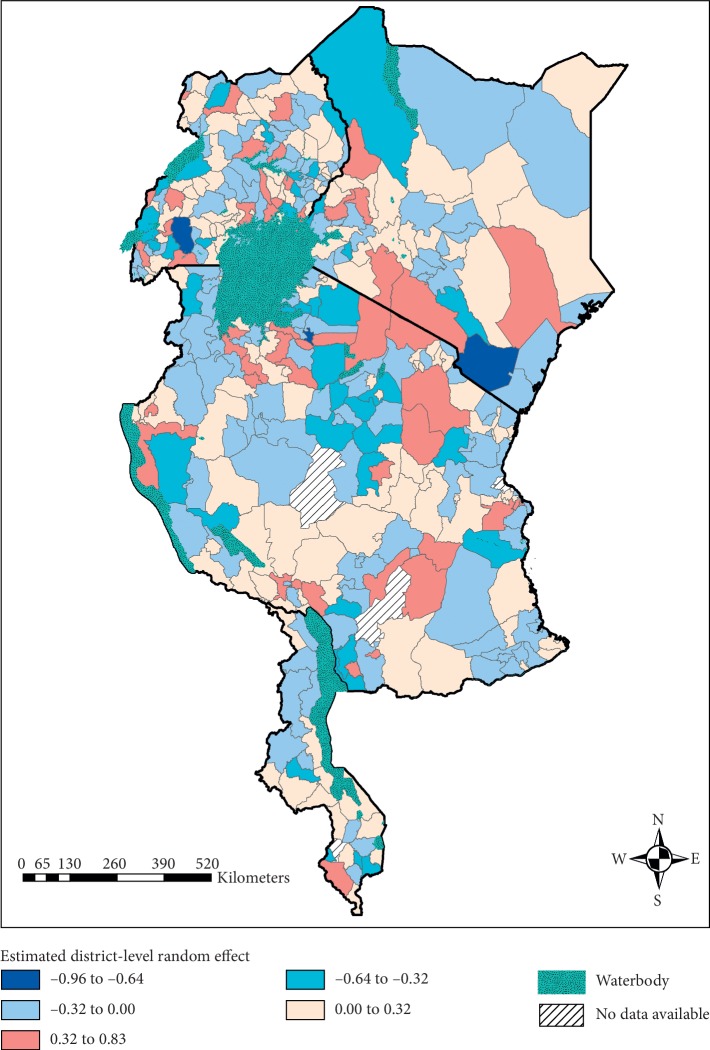
Estimated district-level random effect on the log-odds of anaemia (top left: Uganda; top right: Kenya; middle: Tanzania; bottom: Malawi).

Figure 5 displays the estimated district-level random effect based on the BLUP estimates, where the shades of blue had a negative/decreased effect on the log-odds of anaemia and the shades of beige to red had a positive and therefore increased effect on the log-odds of childhood anaemia. There was significant heterogeneity between and within the countries, with each country consisting of districts with both positive and negative effects. Kenya, Tanzania, and Uganda each contained an isolated district with a considerably lower negative effect. Unlike the cluster-level spatial effect, Malawi displayed significant heterogeneity in this district-level random effect, with one district displaying a notably higher positive effect compared to the rest of the country.

**Figure 6:**
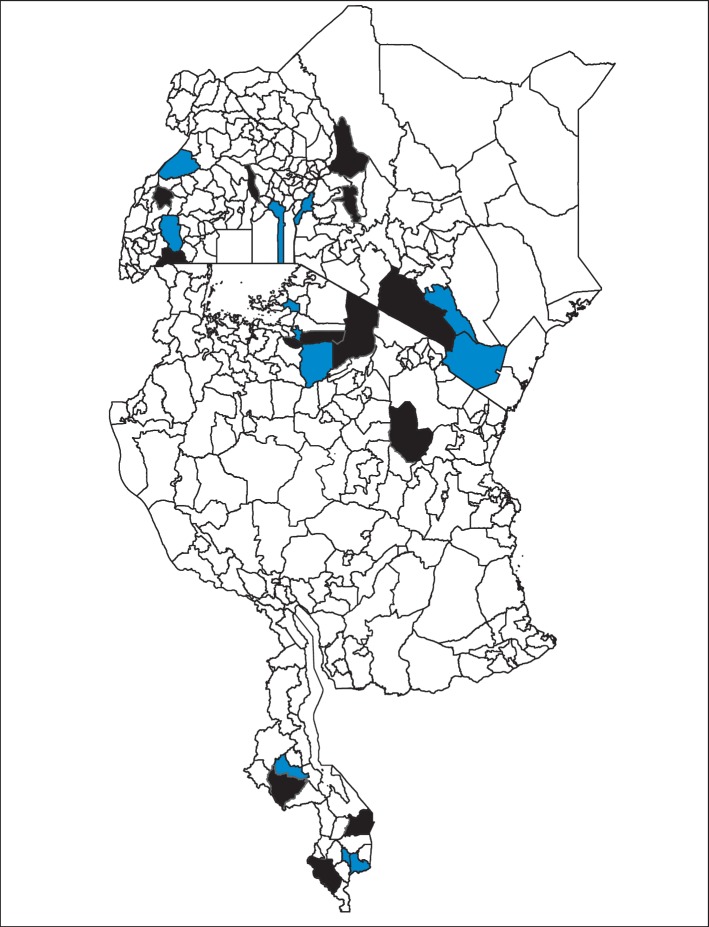
Top 3 districts within each country performing the best (in blue) and the worst (in black) with regard to the odds of anaemia in children (top left: Uganda; top right: Kenya; middle: Tanzania; bottom: Malawi).

Based on the standardised BLUP estimates, the districts were ranked. A negative BLUP is associated with decreased odds of anaemia in the district, while a positive BLUP is associated with increased odds of anaemia in the district. The top 3 “best” performing districts (those with the lowest standardised BLUP values) and the top 3 “worst” performing districts (those with the highest standardised BLUP values) were determined for each country (Figure 6). The best-performing district or county in Kenya was Taita-Taveta County, in Malawi was Mulanje, in Tanzania was Bariadi, and in Uganda was Kiruhura. However, the worst-performing district or county in Kenya was West Pokot County, in Malawi was Chikwawa, in Tanzania was Ngorongoro, and in Uganda was Kyenjojo.

## 4. Discussion

Based on the structure of the surveys and data used in this study, a generalised additive mixed model was employed to assess the association between a child's anaemia status and potential individual, household, and community-level risk factors in Kenya, Malawi, Tanzania, and Uganda while accounting for spatial heterogeneity of childhood anaemia. The study revealed significant spatial heterogeneity of childhood anaemia within and between the districts of the four countries. Two sources of spatial heterogeneity were accounted for, that due to spatial dependence of the observations between the sampled clusters and that due to district-specific factors via the inclusion of a random effect based on the district of residence. The random and spatial effects are surrogates for influences of unmeasured factors, which may be local (district specific) or global (common between neighbouring clusters or districts), respectively [13].

In this study, the heterogeneity in the district-specific random effect suggests that there are local unobserved factors within each district contributing to anaemia in children. A further benefit of adding the district of residence as a random effect is that it allows for the ranking of the performance of the districts on the log-odds of anaemia based on the BLUP estimates, after controlling for potential risk factors of anaemia and spatial autocorrelation [33]. In other words, the BLUP values can be regarded as the estimated effect that a district has on the log-odds of anaemia due to unmeasured factors. It would not have been possible to rank the performance of the districts if the district of residence was added as a fixed effect, which would have resulted in 369 indicator variables for the 370 districts in the model. Not only does this ranking procedure allow for the worst-performing districts to be targeted in order to improve their anaemia control strategies, but it also allows for the best-performing districts to be identified in order to further determine why they are performing better and then to use these districts as examples in efforts to overcome childhood anaemia.

The cluster-level spatial effect allows one to observe any spatial dependence or heterogeneity within the districts of the countries, where many of the districts had more than one sampled cluster. An advantage of incorporating this spatial effect at a cluster level rather than at a district level is that a district-level spatial effect aggregates the effect of spatial autocorrelation, which may result in missing some important information. This was evident by some districts within Kenya, Uganda, and Tanzania containing clusters associated with both a lower and a higher risk of childhood anaemia. This is a clear indication that strategies for anaemia control should be tailored to what is happening within a specific district.

After accounting for the apparent spatial heterogeneity, child-level characteristics (gender, malaria RDT result, and mother's highest education level), household-level characteristics (household size, household's wealth index Z-score, the type of toilet facility available, and the type of place of residence), and the country of residence were found to be significantly associated with the child's anaemia status. These findings are generally in agreement with that in the literature [2, 8, 10, 12, 14, 38–40]. Female children were less likely to be anaemic compared to males. Children with malaria were associated with significantly higher odds of anaemia compared to those that did not have malaria, which is unsurprising as anaemia is a clinical consequence of malaria [41]. Young children are particularly vulnerable as they are yet to build up immunity to malaria, whether in a setting of higher or lower malaria transmission [41]. There were increased odds of anaemia in children with less educated mothers. Besides education being associated with one's earning potential, educated individuals are also associated with the ability to have more awareness and understanding of health- and nutrition-related issues. Furthermore, the odds of anaemia also increased with a decrease in the household's wealth index Z-score. Individuals with low wealth are often subjected to economic constraints where they are not able to afford the dietary and sanitation needs of themselves and their family. Moreover, children in households with no toilet facilities were associated with significantly higher odds of anaemia. Poor sanitation can aid in the development of a number of infectious and parasitic diseases, which indirectly contribute to childhood anaemia [42]. This study revealed increased odds of anaemia in children aged 6 to 10 months. As children in this age group experience accelerated growth during that stage of their lives, they can be more susceptible to anaemia if they do not meet their required nutritional intake of iron-rich food [8].

This study is not without its limits. Due to the cross-sectional nature of the data, the temporal relationship between the child's anaemia status and the covariates could not be determined. Furthermore, the effect of nutritional deficiencies, particularly iron deficiency, on childhood anaemia could not be assessed as no information regarding these factors was available.

## 5. Conclusion

As this study revealed significant evidence of variation between the districts of Kenya, Malawi, Tanzania, and Uganda, further research into the local district-specific drivers of childhood anaemia should be focused on, especially as a one-size-fits-all strategy for anaemia control would not benefit these countries with such spatial variation present. One of the novelties in this study is the introduction of BLUP for accurate assessment and ranking of each district's performance with regard to their impact on childhood anaemia, after controlling for known sources of individual, household, and spatial variation. Based on the best- and worst-performing districts identified in this study, we recommend further investigation into these districts to determine what is unique about them.

## Figures and Tables

**Table 1:** Adjusted odds ratio estimates (AOR) and 95% confidence intervals (CI) for the fixed effects.

	AOR (95% CI)
*Main effects*	
Gender (ref = male)	
Female	0.876 (0.820, 0.935)^*∗*^
Malaria RDT result (ref = negative)	
Positive	4.315 (3.895, 4.781)^*∗*^
Household size	1.014 (1.003, 1.025)^*∗*^
Type of place of residence (ref = urban)	
Rural	0.738 (0.582, 0.936)^*∗*^
Mother's education level (ref = no education)	
Primary	0.843 (0.760, 0.935)^*∗*^
Secondary and higher	0.794 (0.693, 0.911)^*∗*^
Unknown	0.845 (0.742, 0.962)^*∗*^
Gender of household head (ref = male)	
Female	1.016 (0.939, 1.100)
Type of toilet facility (ref = no facilities)	
PIT latrine	0.780 (0.694, 0.877)^*∗*^
Flush toilet	0.725 (0.591, 0.889)^*∗*^
Others	0.663 (0.357, 1.230)
Wealth index *Z*-score	0.847 (0.797, 0.901)^*∗*^
Country (ref = Malawi)	
Kenya	0.316 (0.160, 0.622)^*∗*^
Tanzania	0.639 (0.355, 1.150)
Uganda	0.433 (0.214, 0.874)
Cluster altitude (in 100 metres)	0.986 (0.969, 1.004)
EVI (in 1000 s)	1.026 (0.865, 1.217)
LST	1.015 (0.969, 1.063)

*Interaction effects*	
Type of place of residence and country (ref = urban and Malawi)	
Rural and Kenya	1.376 (1.037, 1.825)^*∗*^
Rural and Tanzania	1.237 (0.942, 1.624)
Rural and Uganda	1.119 (0.826, 1.514)

^*∗*^Significant at 5% level of significance.

**Table 2:** Variance estimates of nonlinear terms.

	Variance estimate
Child's age in months	0.0127
District-level random effect	0.1516
Cluster-level spatial effect	0.6904

## Data Availability

This study utilised existing survey datasets that are in the public domain and freely available from http://www.dhsprogram.com/data/dataset_admin/login_main.cfm with permission from the DHS Program.
